# Creating an intergenerational cohort of mothers and their children using administrative data from health, education and social care in England (ECHILD)

**DOI:** 10.23889/ijpds.v9i5.2498

**Published:** 2024-09-18

**Authors:** Q Feng, Ruth Gilbert, Georgina Ireland, Katie Harron

**Affiliations:** 1 UCL

## Introduction

The linkage of trial data with large administrative databases can enable long-term passive follow-up of participants at a significantly lower cost than direct participant follow-up in a study. Nonetheless, many researchers are unaware of this opportunity and/or the regulatory requirements to facilitate linkage.

## Approach

In 2017, Western University Canada’s Office of Human Research Ethics (OHRE) incorporated a prompt into its online protocol submission platform, asking researchers if they have considered linking their trial data with ICES. ICES is a not-for-profit research and analytics institute in Ontario, Canada with a repository of over 100 data holdings comprised of record-level, coded and linkable health and health-related data. If a researcher selects ‘yes’ to this prompt, they are provided with additional information about ICES, identifiers required for linkage, and language to be included in letters of consent.

## Results

The incorporation of a prompt into the ethics protocol submission platform allowed the OHRE to identify protocols interested in linking trial data with ICES. Over the past 5 years, an average of 2.6% of protocols submitted to the Health Sciences Research Ethics Board included an intent to link data with ICES. The OHRE verified that these protocols had carefully considered regulatory requirements, which helped to streamline the ethical, privacy, and legal processes required for linkage.

## Conclusion

Incorporating a prompt into ethics protocol submission platforms can help to streamline regulatory processes and promote awareness about opportunities to link trial data with large administrative databases.

